# The psychological and social impact of the digital self-support system ‘Brain in Hand’ on autistic people: prospective cohort study in England and Wales

**DOI:** 10.1192/bjo.2023.57

**Published:** 2023-05-26

**Authors:** Samuel Tromans, William Henley, Ian Summers, Danielle Bilkey, Jenna Datson, Nicola Doherty, Louise Morpeth, Sarah Benbow, Rebecca Jelbert, Ashok Roy, Lance Watkins, Bhathika Perera, Saman Shazad, Richard Pender, Regi Alexander, Richard Laugharne, Rohit Shankar

**Affiliations:** Department of Population Health Sciences, University of Leicester, UK; and Adult Learning Disability Service, Leicestershire Partnership NHS Trust, UK; Health Statistics Group, University of Exeter Medical School, UK; Cornwall Intellectual Disability Equitable Research (CIDER), Cornwall Partnership NHS Foundation Trust, UK and University of Plymouth Peninsula School of Medicine, UK; Brain in Hand Ltd, Exeter, UK; Adult Autism Assessment Team, Cornwall Partnership NHS Foundation Trust, UK; Learning Disability Services, Coventry and Warwickshire Partnership Trust, UK; Unit for Development in Intellectual and Developmental Disabilities, University of South Wales, UK; and Mental Health and Learning Disabilities Service, Swansea Bay University Health Board, UK; Learning Disability Services, Barnet, Enfield and Haringey Mental Health NHS Trust, UK; Learning Disability Services, Cheshire and Wirral Partnership NHS Foundation Trust, UK; Autism Services, Devon Partnership NHS Trust, UK; Learning Disability Services, Hertfordshire Partnership University NHS Foundation Trust, UK; and School of Life and Medical Sciences, University of Hertfordshire, UK

**Keywords:** Developmental disorders, autism spectrum disorders, education and training, anxiety disorders, self-harm

## Abstract

**Background:**

Brain in Hand (BIH) is a UK-based digital self-support system for managing anxiety and social functioning.

**Aims:**

To identify the impact of BIH on the psychological and social functioning of adults with autism.

**Method:**

Adults with diagnosed or suspected DSM-5 (level 1) autism, identified by seven NHS autism services in England and Wales, were recruited for a 12-week prospective mixed-methods cohort study. The primary quantitative outcome measures were the Health of the Nation Outcome Scales for People with Learning Disabilities (HONOS-LD) and the Hospital Anxiety and Depression Scale (HADS). Fisher's exact test explored sociodemographic associations. Paired *t*-test was utilised for pre–post analysis of overall effectiveness of BIH. Multivariable linear regression models, univariable pre–post analysis, Wilcoxon signed-rank test, logistic regression analysis, Bonferroni correction and normative analysis were used to give confidence in changes identified. A thematic analysis of semi-structured exist interviews following Braun and Clarke's six-step process of 10% of participants who completed the study was undertaken.

**Results:**

Sixty-six of 99 participants completed the study. There was significant reduction in mean HONOS-LD scores, with 0.65 s.d. decrease in those who used BIH for 12 weeks. Significant positive changes were identified in HONOS-LD subdomains of ‘self-injurious behaviours’, ‘memory and orientation’, ‘communication problems in understanding’, ‘occupation and activities’ and ‘problems with relationship’. A significant reduction in the anxiety, but not depression, component of the HADS scores was identified. Thematic analysis showed high confidence in BIH.

**Conclusions:**

BIH improved anxiety and other clinical, social and functioning outcomes of adults with autism.

## Digital interventions and autism

Autism spectrum disorder (ASD) is a lifelong neurodevelopmental condition, characterised by difficulties in social communication and social interaction, as well as a repertoire of restricted, stereotyped and repetitive behaviours.^1^ Approximately 0.8% of the general population have autism,^2^ with increased prevalence observed among persons utilising mental health services.^3^ Additionally, autistic people are at increased risk of co-occurring mental health conditions compared with their non-autistic peers, with a meta-analysis reporting pooled prevalence estimates of 20% (95% CI 17–23) and 11% (95% CI 9–13) for anxiety and depressive disorders, respectively,^4^ as well as a lower quality of life.^5^ Self-injurious behaviours are also highly prevalent in the autism community, with a recent meta-analysis reporting an overall pooled prevalence estimate of 42% (95% CI 38–47), with a significantly higher rate in females with autism.^6^

## Brain in Hand

There has been significant interest in the use of digital interventions as a means of supporting autistic people.^7^ Such approaches may be of particular benefit to this group because digital interventions provide a predictable and consistent communication and environment, which they can navigate at their own pace, repeating lessons where necessary. Furthermore, digital interventions place reduced social demands on individuals with autism, potentially reducing stress levels compared with in-person interaction.^8^ A meta-analysis of digital interventions for autistic people, including computer programs, tablet apps, a robot and an interactive DVD, reported a small overall effect size, but high heterogeneity between eligible studies.^7^ Furthermore, the majority of studies focussed on children with autism, with a mean participant age across eligible studies of 10.6 years, with less evidence from adults with autism. Additionally, an app designed specifically to manage the anxiety of autistic people by using cognitive behaviour techniques is being trialled.^9^ It has been developed under the principle that autistic people are vulnerable to anxiety, which then affects their daily role and performance. It does not look at whole life function.

Brain in Hand (BIH) is a fusion of digital health solutions for self-management, combining human support in tandem with digital technology, to enable people to live lives that are more independent.^10^ Although designed in partnership with autistic people, it is not condition-specific, and has been implemented in autism and mental health settings.^11^ The BIH tool encourages people to build a personalised support package, with a view to developing their self-management skills, facilitating greater participation in education, employment and community life. This is with a view to increasing the BIH user's confidence to manage daily life challenges, reducing their reliance on statutory services and/or their primary caregiver.

The aim of this study was to identify the strengths and limitations of BIH with respect to the mental health and social functioning for adults with diagnosed or suspected DSM-5^1^ level 1 severity ASD (Supplementary File 1 available at https://doi.org/10.1192/bjo.2023.57), as indicated by the National Institute for Health and Care Excellence (NICE) technological appraisal framework (Supplementary File 2).^12^ The study objectives are detailed in Table 1.

**Table 1:** Quantitative and qualitative study objectives

Quantitative	Qualitative
Measure the impact of BIH on health and social functioning over a 12-week period, using the HoNOS-LD Measure the impact of BIH on mental health, using the HADS Measure the acceptability of BIH, according to study drop-out rates	Understand the participant's experience of using BIH to measure outcomes of reliance on their care support Understand the impact of BIH on coping skills Understand the impact of BIH on the participant's abilities to manage challenging social situations Determine whether BIH helps adults with autism to feel safe Determine whether BIH helps adults with autism to feel more independent Determine whether access to 24/7 telephone support can help autistic people to feel self-empowered to manage their anxiety Determine whether and how the BIH traffic light system helps a person manage their anxiety and independence, and support their coping skills Determine whether participants feel that remote support has improved their access to healthcare during the COVID-19 pandemic

BIH, Brain in Hand; HoNOS-LD, Health of the Nation Outcome Scales for People with Learning Disabilities; HADS, Hospital Anxiety and Depression Scale.

## Method

### Study design

The BIH tool was tested according to 2019 Tier 3A (now Tier C) of the NICE Evidence Standards Framework for Digital Health Technologies (Supplementary File 2), which recommends a large cohort study for the minimum level of evidence to establish the strengths, weaknesses and limitations of any clinical digital technology.^12^ This study employed a mixed-methods cohort design, as neither a qualitative nor a quantitative approach alone would be sufficient to answer the study objectives (Table 1). The Strengthening the Reporting of Observational Studies in Epidemiology (STROBE)^13^ guidance for cohort studies was followed.

The quantitative component involved using the Hospital Anxiety and Depression Scale (HADS)^14^ and the Health of the Nation Outcome Scales for People with Learning Disabilities (HoNOS-LD)^15^ at baseline and 12 weeks post-intervention, to measure changes in anxiety and depressive symptoms, and health and social functioning, respectively. The HADS has been previously validated in a sample of older adolescents and young adults with autism, where it was found to be a reliable and valid measure for this group.^16^ The HONOS-LD has similarly demonstrated good validity and reliability upon testing.^15^ Both tools were chosen after discussion and consideration of a large set of other possible tools. Discussions were had with co-production partners, and scientific and clinical advisors. Strengths, weaknesses, practicality of administration and resources featured in the discussion. More detailed rationale for the selection of all quantitative tools is provided in Appendix 1.

Semi-structured interviews (Supplementary File 3) were completed remotely for those who finished the study, transcribed and differences reconciled before conducting a thematic analysis following Braun and Clarke's six-step process.^17^ Both deductive and inductive approaches were used to generate themes, reflecting on both prior knowledge of the researchers and the development of themes that naturally emerged from the data. The process allowed in-depth conversations between the participant and researcher to gather perception, opinion, experience and emotion. Thematic analysis drew from grounded theory and phenomenological approaches for considering and analysing data.^18^ The qualitative semi-structured interview methodology helped gain an in-depth insight from participants on the study topic.

### Participants

A purposive sampling approach was employed, whereby members of clinical care teams accessed their patient's medical records and autism diagnostic service waiting lists to identify potential participants.

Inclusion and exclusion criteria for the study are summarised in Table 2. A target sample size of 100 participants satisfying eligibility criteria was prespecified. This allowed for a 10% drop-out rate, as a sample size of 90 participants would provide 80% power at a statistical significance level of 5%, to detect a standardised effect size of 0.3 for the change in HADS score^13^ from baseline to 12-week follow-up. This represents a small-to-medium effect size as defined by Cohen.^19^ In the resultant study, the sample size was 99 participants.

**Table 2:** Inclusion and exclusion criteria

Inclusion criteria	Exclusion criteria
Having been diagnosed with autism spectrum disorder as per DSM-5 level 1 criteria, or after screening by a health professional (e.g. general practitioner) and on the confirmatory diagnostic pathway	Any acute or chronic condition, particularly neurodevelopmental conditions such as significant intellectual disability, or level 2/3 DSM-5 autism spectrum
Aged 19–80 years	Aged <19 or >80 years
Screened by the Columbia-Suicide Severity Rating Scale as not having risk concerns of suicide	Anyone who screened positive on the Columbia-Suicide Severity Rating Scale
Access to smart devices with compatibility to run BIH, such as mobile smartphones, tablet devices and laptop computers	Not willing to engage with a smart device/ the internet
Has capacity to give informed consent for study participation	Anyone who declines to give consent or is unable to give informed consent
	Suspected or clinically diagnosed co-occurring mental health conditions (psychosis, severe depression, etc) that would limit the ability of the participant to take part in the study
	Insufficient English language to understand and complete questionnaires

The qualitative group sample size was ten participants, all randomly selected from the quantitative sample, as a systematic review^20^ has reported that saturation can be achieved with interview samples of 9–17 participants, and a smaller sample size would be reasonable considering the comparatively modest claims of this study (rather than for a study of BIH in both autistic and non-autistic groups, or both child and adult groups).^20,21^ Randomisation was performed with an online randomisation tool.^22^ The authors conducting qualitative analysis were I.S., D.B., J.D. and S.B. No statistical analysis was performed in relation to interrater reliability.

Study participants were recruited from seven National Health Service (NHS) healthcare trusts across England and Wales, with a total catchment population of approximately 7 million.

### Patient and public involvement

The participant-facing study documentation (including participant information sheets, consent forms and semi-structured interview questions) was developed in collaboration with the lead NHS site council accessibility team with lived experience of autism. Autistic people were actively involved in examining the design of the study and ensuring the study information and interview questions were accessible. Additionally, BIH had an independent user panel comprising 14 autistic people, who were involved in development of the technology and also provided oversight of the study.

### Analysis

Associations between sociodemographic factors and whether there were differences in participants who had a confirmed autism diagnosis versus those suspected of having an autism diagnosis were assessed with Fisher's exact test. The overall effectiveness of BIH was assessed in pre–post analyses by comparing the mean outcomes in the cohort from baseline to 12 weeks, using a paired *t*-test (presented with 95% confidence intervals for the estimated change in outcomes). Multivariable linear regression models were used to determine whether changes in outcomes were associated with demographic factors, adjusting for baseline outcome scores.

Four models were prespecified to explore factors associated with changes in total HONOS-LD scores (joint primary outcome). The first model adjusted for regression to the mean by including baseline HoNOS-LD score as a covariate. The second model compared HoNOS-LD scores in adults receiving support with those not receiving support, after adjustment for baseline HoNOS-LD score and any significant sociodemographic factors, such as age and employment status. The third added measures of BIH engagement and the fourth added the effect of a confirmed autism diagnosis. Univariable pre–post analysis was conducted to assess the effect of the intervention on each component of the HoNOS-LD. Because of the non-normality of the component scores, the Wilcoxon signed-rank test was used to compare median HoNOS-LD component scores at baseline and 12-week follow-up. A similar approach was taken to assessing change at follow-up for seven specific clusters within the HoNOS-LD measure. Bonferroni correction was conducted to account for multiple testing in the secondary analyses; for example, a more stringent *P*-value threshold of *P* < 0.003 was used in place of the usual *P* < 0.05 when assessing changes in individual HoNOS-LD component scores. Reasons for missing data were documented, and the baseline characteristics of those with and without missing data compared. Logistic regression analysis was conducted to identify demographic and baseline characteristics associated with risk of drop-out. A similar structured approach was adopted for the multivariable analysis of the HADS anxiety and depression scales.

### Ethical and other governance approvals

The authors assert that all procedures contributing to this work comply with the ethical standards of the relevant national and institutional committees on human experimentation and with the Helsinki Declaration of 1975, as revised in 2008. All procedures involving human patients were approved by the NHS Research National ethics committee (NHS Health Research Authority) on 1 June 2021 (reference: 21/SW/0066). Full ethical approval was obtained. BIH was utilised after meeting the NHS requirements of the full digital technology assessment criteria and on passing the Penetration Test, which is an authorised simulated attack performed on the computer system to evaluate its security. Written informed consent was taken from the participant, recorded, countersigned by the researcher and a copy given to the participant. The study was registered with Clinicaltrials.gov under identifier NCT05468541. The first participant was recruited on 6 September 2021, the final participant was recruited on 31 December 2021, and the study end date was 31 March 2022.

## Results

One hundred and one people consented to the study. Two people were found not to meet the inclusion criteria and were excluded before their commencement of the study. The study cohort (*n* = 99), summarised in Table 3, included 52 adults with a formal autism diagnosis (representing 53% of the cohort) and 47 adults on the waiting list for an autism assessment at the time of recruitment. Participant ages ranged from 19 to 80 years. There were no statistically significant (*P* < 0.05) differences between participants with a formal autism diagnosis and the waiting list subgroups with respect to age distribution, employment status, accommodation and support.

**Table 3:** Characteristics of the study population

Variable	On waiting list (*n =* 47)	Diagnosed with autism (*n =* 52)	Overall (*n =* 99)	*P*-value
Gender				
Male	13	15	28	
Female	33	34	67	
Other^a^	1	3	4	
Age group, years				0.67
19–30	19	20	39	
31–40	11	13	24	
41–50	5	5	10	
51–60	10	9	19	
61–80	1	5	6	
Missing	1	0	1	
Employment status				0.85
Employed	21	26	47	
Unemployed	19	19	38	
Other^b^	7	7	14	
Support				1.00
Supported	15	17	32	
Not supported	30	32	62	
Other^c^	2	3	5	
Accommodation				0.31
Own home	20	17	37	
Rented	21	31	52	
Other^d^	6	4	10	
Relationship status				0.90
Married/partner	25	25	50	
Divorced	3	3	6	
Single	18	22	40	
Not known	1	2	3	

a. Including transgender, non-binary and gender non-conforming participants.

b. Including participants in full-time education, as well as those working in a voluntary capacity.

c. Including those supported by a friend, as well as those in receipt of student/educational support.

d. Including participants living with their parents, or in student accommodation.

### Analysis of participants who dropped out

**Fig. 1:**
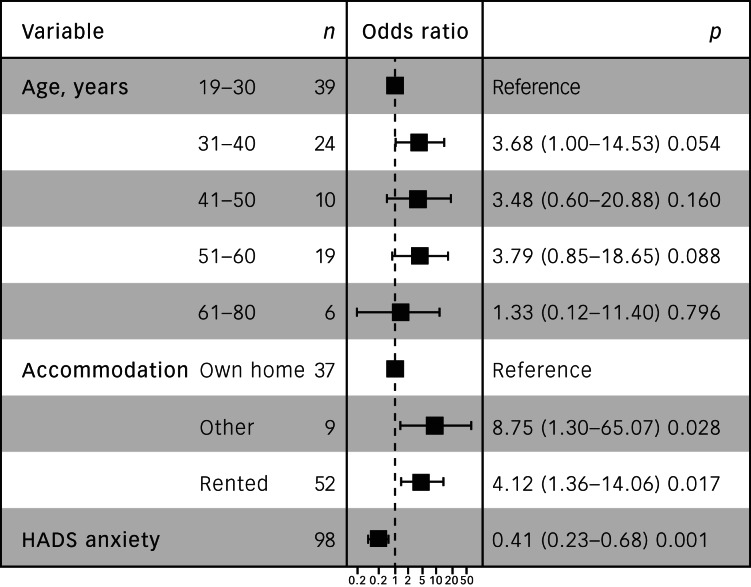
Factors associated with study drop-out. HADS, Hospital Anxiety and Depression Scale.

A total of 33 participants dropped out of the study. Subanalysis of this group showed three factors were associated with study drop-out. This included being in the 31–60 years age brackets and living in rented/other accommodation (Fig. 1). Additionally, participants with higher HADS anxiety scores were at reduced risk of drop-out compared with their peers with lower anxiety scores.

### Quantitative findings

#### HADS

**Fig. 2:**
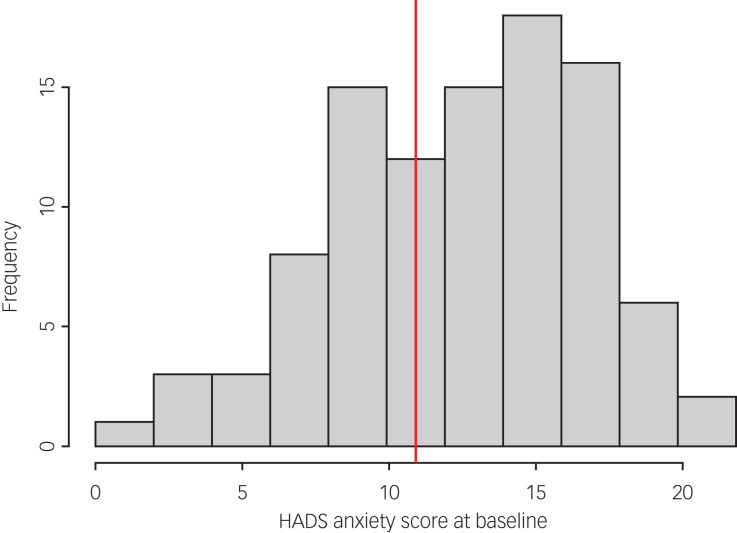
Distribution of HADS anxiety scores at baseline. HADS, Hospital Anxiety and Depression Scale. The vertical red line denotes the mean baseline score.

**Fig. 3:**
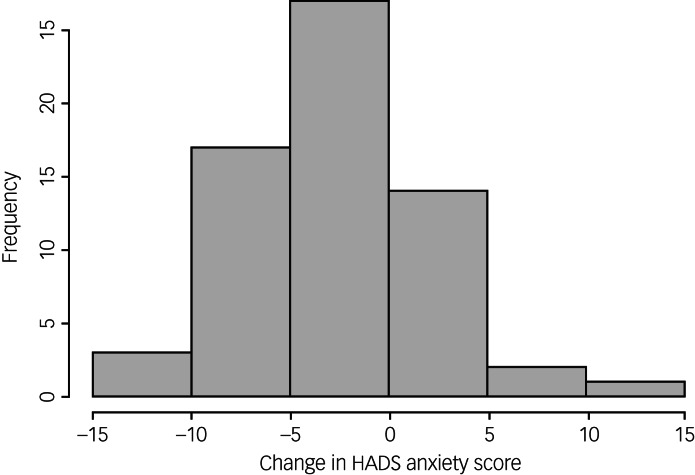
Distribution of change in HADS anxiety scores at follow-up (compared with baseline scores). HADS, Hospital Anxiety and Depression Scale.

**Fig. 4:**
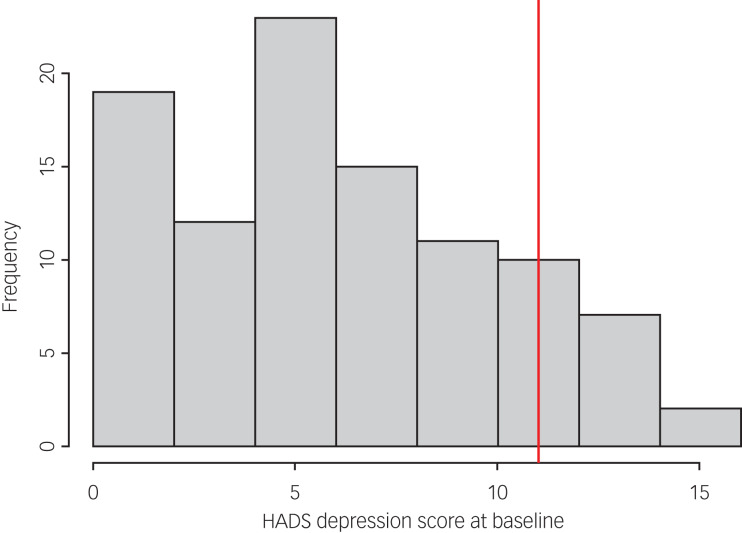
Distribution of HADS depression scores at baseline. HADS, Hospital Anxiety and Depression Scale. The vertical red line denotes the mean baseline score.

Of the 99 study participants, 66 completed the HADS measure at follow-up. A paired *t*-test demonstrated a statistically significant reduction in HADS anxiety scores at follow-up compared with baseline scores (mean reduction −2.23; *P =* 0.0004; 95% CI −3.43 to −1.04). Figure 2 illustrates the distribution of HADS anxiety scores at baseline and Fig. 3 shows the distribution of change in scores among participants. However, there was not a statistically significant change in HADS depression scores at follow-up compared with baseline scores (mean reduction −0.59; *P =* 0.31; 95% CI −1.75 to 0.56). Figure 4 illustrates the distribution of HADS depression scores at baseline.

#### HONOS-LD

Table 4 illustrates the total HONOS-LD scores at baseline and follow-up, in addition to item and item cluster scores. Participants completing the HONOS-LD at baseline (*n =* 99) had a mean score of 18.7 (s.d. 7.6), compared with a mean score of 13.9 (s.d. 8.0) for participants who completed the HONOS-LD at follow-up (*n* = 66). For participants who had completed both baseline and follow-up HONOS-LD questionnaires (*n =* 64), a mean reduction of 5.7 in total scores was observed, which was a significant difference (*P* < 0.001; 95% CI −7.8 to −3.5), indicating an increase in health and social functioning among this group.

**Table 4:** Summary of total, item and item cluster scores at baseline and follow-up

Item	Mean (s.d.)	95% CI	*P-*value
Total HoNOS-LD score			
Baseline (*n =* 99)	18.7 (7.6)		
Follow-up score (*n =* 66)	13.9 (8.0)		
Change score (*n =* 64)	−5.7 (8.7)	−7.5 to −3.5	H0 = 0: < 0.001 H0 = 2.4^a^: 0.004
HoNOS-LD item	Baseline mean	Follow-up mean	*P*-value
1. Behaviour towards others	0.89	0.85	0.37
2. Self-injurious behaviour	1.30	0.58	<0.001
3. Other mental and behavioural problems	1.57	1.42	0.16
4. Attention and concentration	1.25	1.05	0.08
5. Memory and orientation	0.88	0.47	<0.001
6. Communication problems in understanding	1.00	0.39	<0.001
7. Communication problems in expression	0.86	0.59	0.02
8. Hallucinations and delusions	0.09	0.08	1.00
9. Mood changes	1.63	1.38	0.02
10. Sleep problems	1.66	1.39	0.04
11. Problems with eating and drinking	1.04	0.68	<0.001
12. Physical problems	0.62	0.62	0.73
13. Seizures	0.07	0.06	0.37
14. ADL at home	1.18	0.94	0.11
15. ADL outside home	1.22	1.05	0.04
16. Level of self-care	0.86	0.71	0.16
17. Problems with relationships	1.53	0.89	<0.001
18. Occupation and activities	1.12	0.74	<0.001
Item cluster	Baseline median	Follow-up median	*P-*value
Behavioural problems (items 1–3)	5.0	3.5	0.001
Cognition (items 4 and 5)	2.0	1.5	0.003
Communication (items 6 and 7)	2.0	1.0	0.001
Mental state (items 8–11)	4.0	4.0	<0.001
Physical problems (items 12 and 13)	0.0	0.0	0.61
ADL (items 14–16)	3.0	2.0	0.01
Social functioning (items 17 and 18)	2.0	1.0	<0.001

HoNOS-LD, Health of the Nation Outcome Scales for People with Learning Disabilities; ADL, activities of daily living.

a. H0 = 0: the *p*-value based on a null hypothesis of no change in total HoNOS-LD score. H0 = 2.4: the *p*-value based on a null hypothesis of a mean reduction in total HoNOS-LD score of 2.4, as previously reported by Roy et al.^15^

Four models were used to explore the factors that might be associated with any major variance in HONOS-LD scores. Model 1, adjusted by including baseline HONOS-LD score as a covariate to address regression to the mean, estimated the change from baseline to follow-up as a reduction of 5.3 in HONOS-LD scores. Model 2 showed an estimated mean reduction of 7.8 points for those not receiving support, but a lesser reduction of 1.5 points for those who received support (*P* = 0.006); no other sociodemographic factors were significantly associated with a change in total HONOS-LD score. Model 3 adjustment did not demonstrate any variation in total HONOS-LD score reduction from baseline to follow-up, according to measures of BIH app engagement. Model 4 found no effect of autism diagnosis on the overall reduction in HONOS-LD scores. Expected HONOS-LD scores reduced by 0.65 s.d. following the BIH intervention.

Additionally, a normative analysis was conducted by comparing mean reductions in total HONOS-LD scores with those reported in a previous 12-week validation study of the HONOS-LD (*n* = 372) that assessed its reliability and sensitivity to change.^14^ The mean reduction in HONOS-LD total score following the BIH intervention was significantly greater than expected based on the null hypothesis of a 2.4-point reduction from the normative study (*P* = 0.004).^14^

Table 4 summarises the scores for specific HONOS-LD items and item clusters. Of note are the statistically significant (*P* < 0.003) reductions in scores for self-injurious behaviours, memory and orientation, communication problems in understanding, problems with eating and drinking, problems with relationships, and occupation and activities items. Other items, including communication problems in expression, mood changes, sleep problems and activities of daily living outside the home, showed significant decreases, but did not meet the threshold for the Bonferroni correction. Statistically significant reductions in item cluster scores were observed for all item clusters except physical problems (items 12 and 13).

### Qualitative findings

All ten study participants randomly selected for interview consented to participate. The demographic details of the study participants are summarised in Table 5. Thematic analysis of the interviews was conducted. Verbatim comments relating to each of the themes is provided in Table 6. Data saturation was achieved.

**Table 5:** Demographic details of the participant group who underwent semi-structured qualitative interviews (*n =* 10)

Variable	*n*
Status at time of study participation	
Autistic	5
Waiting list	5
Gender	
Male	4
Female	6
Age group, years	
19–30	5
31–40	3
41–50	1
51–60	1
Employment status	
Employed	7
Unemployed	2
Other^a^	1
Support	
Supported	5
Not supported	5
Accommodation	
Own home	3
Rented	6
Other^b^	1
Relationship status	
Married/partner	6
Single	4

a. Including participants in full-time education, as well as those working in a voluntary capacity.

b. Including participants living with their parents, or in student accommodation.

**Table 6:** Themes identified from participant interviews, with corresponding participant quotes

Themes	Quotes
Set up process of Brain in Hand (on-boarding)	‘It could have quite easily of been condensed.’
Building confidence	‘At supermarkets where I get overwhelmed, for example, even if I didn't use it, it just gave me that little bit of extra confidence I think.’ ‘Planning is the biggest thing for me.’ ‘I've been mainly using my parents for symptoms of anxiety.’
Traffic light system and self-awareness	‘Because I don't understand my feelings that well, the traffic light system has been a blessing.’ ‘I've mostly used, um, the traffic lights system just to keep track of my mood.’
Suggested developments by participants	‘I'm just gonna use the red traffic light and just the act of doing that kind of pulled me out of that cycle.’ ‘I have to program everything in manually and then analyse it, whereas if I could just upload my calendar to it then I would find that a lot easier.’ ‘Unfortunately, on the app you can only see daily, so it's not very helpful when you're trying to plan a week.’
Recommending to other autistic people	‘I would recommend it to people … I think while people are on the waiting list - Brain in Hand could then be expanded to more of an educational element as well because people will be able to self-manage a lot better without getting as distressed.’
COVID-19 and experience of isolation	‘Loved it.’ ‘Enjoyed lockdown.’ ‘Home was a safe place.’ ‘I quite enjoyed the lockdowns. Um. But I think it's because I didn't have to deal with people.’ ‘I didn't particularly mind being inside. It was more when we started to go out again that the anxiety hit.’

### Set-up process of BIH (on-boarding)

Interview participants expressed positive views about on-boarding, particularly when allocated a specialist who also disclosed that they had autism. However, participants expressed that smartphone compatibility with the BIH technology was a recurrent issue. Usability was reported as simple, but some participants expressed a need for an information technology (IT) ability assessment for all BIH participants, as some participants experienced frustration when their high level of IT ability was not recognised during the on-boarding process.

### Building confidence

Participants described BIH as having a positive effect on their confidence. Participants also emphasised the importance of building a partnership with their specialist, where personalised support aided a development of self-awareness relating to their autism and how it affects them. Participants also felt the function of the digital health tool to support around unplanned events with manageable steps was important in mitigating sensory overload. They also discussed how the digital health tool enhanced their independence by providing an additional support option if needed, reducing their reliance on others for support.

### Traffic light system and self-awareness

Participants reflected on how the traffic light system (a simple mood-monitoring tool) supported developments in their own emotional awareness. Participants reported that the reassurance of having access to the traffic light system prevented an escalation of their sensory overload, as it prompted them to stop, identify their emotions and encourage a positive mood.

### Suggested developments by participants

Participants suggested further personalisation during on-boarding, specifically in regard to IT skill level. Participants suggested developments relating to the traffic light system. These include more sophisticated mood tracking, as well as further options when selecting ‘red’ (the mechanism for requesting additional help), as many participants reported anxiety when receiving a call from the response service.

Participants expressed frustration with the calendar feature being unable to synchronise with other apps and technology programs. Additionally, participants reported limitations with the planning tool, including being only able to view one day at a time, and the inability to future plan.

Additionally, participants expressed a desire to be able to access the website immediately. At present, the mobile app is available immediately but the website requires manual login each time, leading to some participants reporting issues with accessibility at times of crisis.

Some participants reported that it would be helpful for the app to have a function whereby they could contact other BIH users to provide peer support to one another. Relatedly, participants suggested that a bank of strategies could be available for BIH users.

Participants living in areas with poor Wi-Fi connectivity sometimes found it difficult to add content to their app. Furthermore, the cost of internet connectivity and IT equipment all need to be considered.

### Recommending BIH to other autistic people

All interview participants reported that they would recommend BIH to another person with autism, with several reporting that they were hopeful that digital health tools could support autistic people in developing self-awareness and aiding education. However, some participants advised that they would recommend BIH with caution, as enhanced commitment is required during set-up to allow the app to be effective. Participants who spent more time personalising the tool experienced more positive outcomes. Three participants felt that BIH should be treated as an addition to, rather than a replacement for, current care.

## Discussion

The primary aim of the study was to identify the strengths and limitations of BIH with respect to the mental health and health and social functioning of adults with autism. Our findings demonstrate a significant increase in overall health and social functioning for participants who used BIH for a 12-week period, as measured by the HoNOS-LD. Additionally, there were significant reductions in scores for numerous HoNOS-LD items, including self-injurious behaviours, memory and orientation, communication problems in understanding, problems with eating and drinking, problems with relationships, and occupations and activities. Additionally, significant reductions in self-reported anxiety were observed, as measured with the HADS.

These findings suggest that when BIH is used for a minimum of 12 weeks, it can improve overall health and social functioning and reduce anxiety symptoms. The overall effect size (0.65) of the effect of the intervention seen on the health and social functioning measures within the HoNOS-LD suggest a strong positive impact on the lives of adults with autism who use BIH. Participants who used BIH for the study period not only benefited from the intervention, but were also satisfied with the overall experience.

Semi-structured interviews with BIH users found that they were better at managing activities of daily living, particularly outside of the home. Multiple participants reported increased confidence and the reassurance of a ‘safety net’, which made challenging situations easier.

BIH can reduce the inequalities and improve the care that adults with autism receive, through a personalised, alternative and creative means of autism support. However, approximately a third (*n =* 33) of the study population dropped out, suggesting that BIH is not suitable for all adults with autism. Factors associated with increased likelihood of drop-out included participants not owning their own house, being over 30 years of age and having lower HADS anxiety scores. These factors could be conceivably explained by some older adults feeling digitally excluded from using the tool, and those with less anxiety feeling that the intervention may be less valuable for them. Additionally, findings from the semi-structured interviews could point to further potential causes of study drop-out, including experiencing problems with on-boarding and issues regarding smartphone compatibility with BIH. Further work needs to be undertaken to better understand factors associated with study drop-out, and how the BIH tool can be modified to address such issues.

### On-boarding and involvement of peers with autism

Adults with autism benefit from personalised support and guidance, which has previously been reported by this group to be both beneficial and desirable.^23^ Evidence from this study highlights that enhanced commitment and personalisation in the set-up process allows the technology to be more effective. Previous digital research has stressed caution and highlighted the importance of recognising the heterogeneity of the autism community, and that not all digital health tools will suit all adults with autism.^24^ BIH somewhat addresses these cautions by permitting the user to connect with a personal autism specialist, to personalise the apps features according to the user's goals. Nevertheless, there were some limitations with the set-up process, highlighting that the increased level of personalisation can cause stress for autistic people, who can find communication and planning difficult. However, previous findings suggest that communication between persons with autism and their autistic peers can be highly effective.^25^ The findings of our study support this assertion, further suggesting the need to consider inclusion of peer support within adult autism services.

### Anxiety

Anxiety disorders are among the most common co-occurring conditions that adults with autism experience.^26^ Our findings demonstrated a significant reduction in anxiety symptoms through use of BIH. Difficulties in communication, social interaction and sensory overload are among the most common predictors of anxiety in individuals with autism.^26,27^ Participant reports from semi-structured interviews suggest that BIH supports goal planning in an effective systematic way, reducing sensory overload and facilitating the ability to utilise coping strategies effectively, to reduce feelings of anxiety and distress. Therefore, BIH might be highly valued among adults with autism, their carers and clinicians.

### Self-injurious behaviour

Self-harm is over three times more common in autistic people compared with their non-autistic peers.^28^ Our findings demonstrated a highly significant reduction in self-harm behaviours among participants using BIH. This suggests that BIH, with its focus on positive coping strategies, is an effective means of reducing self-harm in the adult autism community.

Autistic people experience alexithymia, and improved recognition of one's emotions may support their well-being.^29^ Therefore, by supporting an increase in emotional awareness, the ‘traffic light’ feature on the digital tool has the potential to increase the ability to recognise distress before reaching an intolerable level, where self-injurious behaviours can present.

### Activities of daily living

Adults with autism can find activities of daily living, such as leaving the house, shopping and attending appointments, overwhelming.^30^ This study has demonstrated that the BIH tool has the potential to improve the health and social functioning of adults with autism, supporting independence, socialisation and integration into the community.

### Strengths and limitations

The study cohort was recruited from multiple sites across England and Wales, increasing the generalisability of study findings. Furthermore, adults with autism were actively involved in development of the BIH tool, the study protocol and the study implementation. The mixed-methods study design enabled a richer quality of data collection, reflecting the complexity in supporting adults with autism. However, the sample sizes for both the quantitative and qualitative study samples were relatively small, with the quantitative sample underpowered based on our *a priori* power calculation (which set a target sample size of 90 participants), as only 66 participants completed follow-up. This could have led to the study being unable to detect potentially significant effects that may have been present had the study been fully powered. Furthermore, sample size for grounded theory-based approaches in qualitative research should not be determined *a priori,* as ‘it is contingent on the evolving theoretical categories’.^31^

The study participants comprise adults on autism diagnostic clinic waiting lists as well as adults with autism with level 1 severity according to DSM-5 criteria. It was thought appropriate to include the ‘waiting list group’ because those on the autism waiting list would have a high likelihood of autism, as they would have been screened by a non-autism specialist clinical professional. Our statistical analysis showed that there were no differences between the two groups at baseline. It is also worth noting that there are many undiagnosed adults with autism in the general population who are unaware of their condition.^2^ Ideally, to assess the effectiveness of any intervention on all adults with autism, one would need to conduct active case finding within the general population, to identify previously undiagnosed persons with autism.^32^ This is not practical or feasible.

Additionally, because our study lacked a non-autistic participant group, we cannot be certain that the positive therapeutic effects of BIH are specific to persons with autism. It is possible that other vulnerable groups with similar needs or clinical presentations may experience similar benefits. Additionally, the lack of a randomised control group prevented direct comparison of the BIH intervention with treatment as usual. However, considering the heterogeneous study population and the nature of the intervention being a complex digital tool, a randomised controlled trial without major biases would be challenging. It is worth mentioning that a normative analysis undertaken with previous validation work of the HONOS-LD for the same duration of 12 weeks^15^ showed that the mean reduction in HONOS-LD total score following the BIH intervention was significantly greater than expected, based on the null hypothesis of a 2.4-point reduction from the normative study. However, there are considerable challenges with validity and reliability when using psychometric measures with autistic people that have not been validated in this specific participant group, and as a result, our findings should be interpreted with some caution. A measure of anxiety specifically designed for adults with autism is also available,^33^ which could have been considered as an alternative to the HADS.

An opportunity was missed to collect ethnicity and education data from the study population, which would have permitted subgroup analyses as to whether such factors influence the effectiveness of the BIH intervention and helped to ensure that BIH is a valuable tool for all groups. The study duration was 3 months. It would be useful to see if the findings identified are sustained over a longer time period. Additionally, there may have been other factors influencing self-reported anxiety levels and quality of life that were not controlled for, thus there is a possibility that other factors unrelated to BIH may have contributed to the our findings.

### Implications for clinical practice

BIH has demonstrated effectiveness in improving the health and social functioning of adults with autism, as well as reducing anxiety symptoms and self-harm behaviours. No significant adverse events were reported, and it is considered safe for use.

### Implications for policy

Provision of social and healthcare support is a well-recognised challenge for the autism community.^34^ The findings of the current study support BIH fulfilling the minimum required evidence to meet the research standards for Tier C of the NICE Digital Health Technologies Framework (Supplementary File 2).^12^ It also satisfies the requisite clinical and governance requirements of the Organisation for the Review of Care and Health Apps.^35^ The BIH intervention needs to be subjected to robust economic evaluation.

### Implications for research

More research needs to be done to establish the impact of BIH over time periods exceeding 12 weeks, as well as the impact of adjustments made to accommodate for the needs of adults with autism at greater likelihood of drop-out. Further research needs to be conducted to establish whether BIH could similarly benefit other groups, including those with mental health needs.

## Data Availability

The data that support the findings of this study are available from the corresponding author, R.S., upon reasonable request.

## Supplementary material

For supplementary material accompanying this paper visit http://doi.org10.1192/bjo.2023.57. click here to view supplementary material

## Appendix 1. Study measures used and their rationale

The Columbia-Suicide Severity Rating Scale (C-SSRS)^36^ is a suicide risk assessment that measures suicidal ideation and behaviour across four constructs: severity of ideation, intensity of ideation, behaviour and lethality. It demonstrates good convergent and divergent validity compared with other scales pertaining to suicidal ideation and behaviour, and is suitable for use in both clinical and research settings.^37^ As part of the recruitment process, potential participants were screened with the C-SSRS, as individuals had to be found to have no risks of suicide on this measure to be eligible to participate in the study.

The HADS^14^ is a self-report questionnaire that has been recommended by the NICE to detect symptoms of depression in clinical practice.^38^ It has demonstrated validity and reliability in detecting anxiety and depression in adults within community settings, including in older persons and adults with autism.^16,39–41^

The HoNOS-LD,^15^ a clinician-reported outcome measure, has been found to be a valid and reliable measure of health and social functioning for people with intellectual disability and co-occurring autism and additional mental health needs.^15^ The scale demonstrates certain limitations pertaining to validity and reliability, as previous research suggests concerns relating to behaviour and mood recording.^42^ However, such concerns are not associated with higher-functioning autism.^43^ Compared with the generic HONOS measure,^44^ it contains more items pertaining to behavioural and social functioning, and thus is more appropriate for use with adults with autism.

The interviews^17,18,45,46^ comprised detailed conversations between the research team member and ten participant interviewees, and took place approximately 12 weeks after completion of the baseline questionnaire measures. Interviews were generally of 30–50 min duration, although the timescale was flexible according to participants’ needs.
