# Supplementary material for: The psychological and social impact of the digital self-support system ‘Brain in Hand’ on autistic people: prospective cohort study in England and Wales
Source: BJPsych Open. 2023 May 26;9(3):e96. doi: 10.1192/bjo.2023.57 (PMC10228225; doi:10.1192/bjo.2023.57)
Supplement: Supplementary file 1 [file bjosup.zip › S2056472423000571sup001.docx]

**Supplementary information 1**: Summary of the DSM-5^1^ autism diagnostic criteria (referred to as autism spectrum disorder within DSM-5), as well as the description for level 1 autism severity. For the diagnostic criteria in full, please refer to the source text.

| **DSM-5 Criteria for Autism Spectrum Disorder:**   1. Persistent deficits in social communication and social interaction across multiple contexts 2. Restricted, repetitive patterns of behaviour, interests, or activities 3. Symptoms must be present in the early developmental period 4. Symptoms cause clinically significant impairment in social, occupational or other important areas of current functioning 5. These disturbances are not better explained by intellectual disability (ID)^^[[1]](#footnote-1)^^ |
| --- |
| **Level 1 Severity (“Requiring Support”) Description:**   1. **Social communication:** Without supports in place, deficits in social communication cause noticeable impairments. Difficulty initiating social interactions, and clear examples of atypical or unsuccessful responses to social overtures of others. May appear to have decreased interest in social interactions. 2. **Restricted, repetitive behaviours:** Inflexibility of behaviour causes significant interference with functioning in one or more contexts. Difficulty switching between activities. Problems of organisation and planning hamper independence. |

1. DSM-5 recognises that ASD and ID frequently co-exist, but that “to make comorbid diagnoses of autism spectrum disorder and intellectual disability, social communication should be below that expected for general developmental level.” [↑](#footnote-ref-1)
