# Supplementary material for: The psychological and social impact of the digital self-support system ‘Brain in Hand’ on autistic people: prospective cohort study in England and Wales
Source: BJPsych Open. 2023 May 26;9(3):e96. doi: 10.1192/bjo.2023.57 (PMC10228225; doi:10.1192/bjo.2023.57)
Supplement: Supplementary file 1 [file bjosup.zip › S2056472423000571sup002.docx]

**Supplementary information 2:** Comparison of BiH intervention to the NICE Digital Health Technologies Framework (level C).

| **Evidence category** | **Nature of minimum accepted evidence** | **BiH research study findings** |
| --- | --- | --- |
| Demonstrating effectiveness for preventative behaviour change or self-manage functions | High-quality observational or quasi-experimental studies demonstrating relevant outcomes. These studies should present comparative data. Comparisons could include:   - Relevant outcomes in a control group - Use of historical controls - Routinely collected data.   Relevant outcomes may include:   - Behavioural or condition-related user outcomes such as reduction in smoking or improvement in condition management - Evidence of positive behaviour change - User satisfaction. | Cohort study with a normative analysis to original validation of the used health and social functioning tool.  Demonstrated significant reductions in overall HONOS-LD scores and levels of Anxiety on HADS  User satisfaction of those who competed the study was very high |
| Use of appropriate behaviour change techniques (if relevant) | Be able to show that the techniques used in the Digital Health Technology (DHT) are:   - Consistent with recognised behaviour change theory and recommended practice (aligned to guidance from NICE or relevant professional organisations) - Appropriate for the target population. | BiH targets important areas of functioning where autistic adults frequently encounter difficulties. It help planning and decision-making and managing anxiety.  There was recognition of significant change on use of BiH in these symptom profiles for autistic adults. |
| Reliable information content | Be able to show that any health information provided by the DHT is:   - Valid (aligned to best available sources, such as NICE guidance, relevant professional organisations or recognised UK patient organisations, and appropriate for the target population) - Accurate - Up to date - Reviewed and updated by relevant experts at defined intervals, such as every year - Sufficiently comprehensive | BiH is level 2 certified by the Organisation for the Review of Health and Care Apps (ORCHA), meaning that it is focussed on general health.^41^ |
