# Supplementary material for: The psychological and social impact of the digital self-support system ‘Brain in Hand’ on autistic people: prospective cohort study in England and Wales
Source: BJPsych Open. 2023 May 26;9(3):e96. doi: 10.1192/bjo.2023.57 (PMC10228225; doi:10.1192/bjo.2023.57)
Supplement: Supplementary file 1 [file bjosup.zip › S2056472423000571sup003.docx]

**Supplementary information 1:** Semi-structured interview template.

**Brain in Hand**

**Semi Structured Questions**

Date:……/……./ 2021

Interviewer: …………. Participant ID Code: (……………) Site ID Code (……..)

In person: (Telephone) (Video) (Audio)……………………….

Start Time:……………. End Time:…………….

1. **Using Brain in Hand:**

**Q)** What was your experience of using Brain in Hand?

**Prompts:**

- Can you describe how confident you felt using Brain in Hand?
- Can you tell me how long it took you to feel confident in using Brain in Hand?
- What was your experience of the one to one support you received in using Brain in Hand?

1. **Plan your Day Experience:**

**Q)** Can you tell me how Brain in Hand has helped you plan your day?

**Prompts:**

- Do you feel more able to plan your day when using Brain in Hand?
- What do you find difficult in planning your day?
- Can you tell me about a plan for the day that helped you when using Brain in Hand?

1. **Independence:**

**Q)** Can you explain how Brain in Hand has helped you with your independence?

**Prompt:**

- What was your experience of independence when using Brain in Hand?
- Can you talk about a time when Brain in Hand helped you to feel more independent?
- Can you explain if you have relied on family, friends or others for support before using the Brain in Hand App and how this might have changed?
- Can you tell me about a time when you used the Brain in Hand App to support your independence, which previously you would have used family, friend or other to support you or from other services?

.

1. **Coping Skills Experience:**

**Q)** Can you tell me how you coped with change by using your coping skills from the Brain in Hand App?

**Prompt:**

- Tell me about your experience of using your coping skills from the Brain in Hand App?
- What differences in your coping skills have you noticed when using the Brain in Hand?
- Tell me how you would cope with change if you did not have the Brain in Hand App?

1. **Anxiety experience:**

**Q)** What is your experience of anxiety when using Brain in Hand?

**Prompt:**

- Can you tell me how Brain in Hand has helped you when you felt anxious?
- Can you explain how your anxiety felt before you used the Traffic Light System?
- Can you explain how your anxiety felt after seeking support from the Traffic Light System?
- What was your experience of receiving support from Brain in Hand App when feeling anxious?
- When using Brain in Hand App, can you explain any differences in your anxiety?
- If you did not have the Brain in Hand App, how would you cope with your anxiety? Would you seek support from other services that you now don’t need to?
- How has Covid 19 isolation affected your anxiety?

1. **Mood Experience:**
2. How would you describe your mood after using Brain in Hand App?

**Prompt:**

- How would you describe your mood today?
- How would you describe your mood before using Brain in Hand?
- Can you explain how your mood has been supported when using Brain in Hand?
- Can you give me an example of when Brain in Hand helped you cope with a low mood?
- Can you explain how you would cope without using the Brain in Hand App?
- Can you explain if Covid 19 has impacted on your mood and the use of Brain in Hand App?

1. **New Activities and Opportunities:**

**Q)** Can you tell me about any new activities or opportunities you have started in the last three months?

**Prompt:**

- What was your experience of attending a new activity with Brain in Hand support?
- Can you explain how Brain in Hand has supported you when going to new activities or opportunities?
- Would you be able to start new activities without support and could you explain what support you would need to attend an activity?
- What was your experience of accessing new activities during Covid 19?

1. **Experience of Using Telephone Support?**

**Q)** Can you discuss your experience of accessing the Brain in Hand telephone support line?

**Prompt:**

- Tell me how you felt when receiving telephone support?
- Can you explain how having access to a 24 hour helpline has helped you? What might you have done if you couldn’t have called the helpline?
- Can you explain any difficulties you experienced in accessing telephone support?

1. **Benefits of using Brain in Hand:**
2. What do you feel has benefited you most in using Brain in Hand?

**Prompt:**

1. My Anxiety.
2. My Planning Skills.
3. My Coping Skills.
4. My Mood.
5. My Problem Solving.
6. My ability to cope better.
7. My Access to 24 hour Support.
8. My Independence.
9. **How would you describe your life now: if you did not have the opportunity to use the Brain in Hand App?**
10. **What improvements would you make to Brain in Hand and would you recommend the Brain in Hand App to other autistic people?**
